# Identification of a Sensitive Human Immunological Target of Aryl Hydrocarbon Receptor Activation: CD5^+^ Innate-Like B Cells

**DOI:** 10.3389/fimmu.2021.635748

**Published:** 2021-04-15

**Authors:** Lance K. Blevins, Jiajun Zhou, Robert B. Crawford, Norbert E. Kaminski

**Affiliations:** ^1^ Institute for Integrative Toxicology, Michigan State University, East Lansing, MI, United States; ^2^ Department of Microbiology & Molecular Genetics, Michigan State University, East Lansing, MI, United States; ^3^ Department of Toxicology & Pharmacology, Michigan State University, East Lansing, MI, United States; ^4^ Center for Research on Ingredient Safety, Michigan State University, East Lansing, MI, United States

**Keywords:** human, innate-like B cells, AHR, PD-1, immune suppression

## Abstract

Xenobiotic-mediated activation of the aryl hydrocarbon receptor (AHR) is immunotoxic in a number of immune cell types, with the B cell being a well-established sensitive target. Recent advances have provided evidence that the B cell repertoire is a heterogeneous population, with subpopulations exhibiting vastly different cellular and functional phenotypes. Recent work from our laboratory identified the T cell specific kinase lck as being differentially regulated by *2,3,7,8-tetrachlorodibenzo-p-dioxin* (TCDD), which is a potent activator of AHR. While LCK is primarily expressed in T cells, a subset of CD5^+^ B cells also express LCK. CD5 positivity describes a broad class of B lymphocytes termed innate-like B cells (ILBs) that are critical mediators of innate immunity through constitutive secretion of polyvalent natural immunoglobulin M (IgM). We hypothesized that CD5^+^ ILBs may be sensitive to AHR-mediated immunotoxicity. Indeed, when CD5^+^ B cells were isolated from the CD19^+^ pool and treated with TCDD, they showed increased suppression of the CD40 ligand-induced IgM response compared to CD5^-^ B cells. Further, characterization of the CD5^+^ population indicated increased basal expression of *AHR*, AHR repressor (*AHRR*), and cytochrome p450 family 1 member a1 (*CYP1A1*). Indeed the levels of AHR-mediated suppression of the IgM response from individual donors strongly correlated with the percentage of the B cell pool that was CD5^+^, suggesting that CD5^+^ B cells are more sensitive to AHR-mediated impairment. Together these data highlight the sensitive nature of CD5^+^ ILBs to AHR activation and provide insight into mechanisms associated with AHR activation in human B cells.

## Introduction

Aryl hydrocarbon receptor (AHR) is a prototypical xenobiotic sensing receptor/transcription factor that has been extensively studied in the context of toxicological responses to environmental chemical exposure (1, 2). However, in the last decade, research into the biological roles of the AHR in the absence of xenobiotic exposure has increased markedly. In this time, the AHR has been described as a *bona fide* transcription factor critical for the differentiation of type 2 innate lymphoid cells (ILC2) (3). Further, AHR activation can promote the differentiation of T_helper_ 17 (Th17) cells and is necessary for their secretion of Th17 associated cytokines such as IL-17 (4). The AHR has been further implicated in other immune cell subsets as well (5–7).

Despite the abundance of research on the AHR in other immune cell subsets, the AHR has historically been studied in the context of immunotoxicology with B cells representing one of the most sensitive immunological targets of xenobiotic-mediated AHR activation as evidenced by suppression of B cell activation and secretion of immunoglobulins (Ig) (1). Similar to T lymphocytes, it is appreciated that B cells are not a homogeneous lymphocyte population. Yet little research has been conducted to determine if different subsets of B cells are selectively sensitive to AHR activation. Previous work identified lymphocyte-specific protein tyrosine kinase (LCK) as a critical mediator of immunotoxicity in human B cells following treatment with *2,3,7,8-tetrachlorodibenzo-p-dioxin* (TCDD); a high affinity AHR ligand (8). Moreover, TCDD-mediated AHR activation significantly induced expression of LCK in human B cells (8). Our finding was curious as LCK is generally considered to be expressed by T cells, not B cells. This notion has been challenged by the reported finding that CD5^+^ chronic lymphocytic leukemia (CLL) cells and their CD5^+^ B cell progenitors highly express LCK (9–11). CD5 is an immune inhibitory receptor that dampens signaling through the antigen receptor (12, 13). While it is primarily expressed by T cells, subsets of B cells also express CD5 (9–13). While the distribution and specific identity of human CD5^+^ B cells remains controversial, CD5 expressing human B cells are loosely termed ‘innate-like’ B cells (ILBs; IBCs) (14–16). Hence we hypothesized that CD5^+^ ILB could be selectively sensitive to AHR-mediated impairment.

ILBs are a heterogenous B cell population, many of which express CD5, that have characteristics similar to murine B1 B cells (14–18). ILBs constitutively secrete polyvalent, natural IgM (nIgM) and are responsible for 80-90% of circulating IgM in the absence of infection or vaccination (14, 15, 17, 18). Given the polyvalent nature of the IgM they secrete, they typically have less mutated B cell receptors, lower affinity IgM, and typically bind non-T-dependent antigens (14, 15, 17, 18). Importantly, ILBs are critical mediators of humoral immunity in neonates when adaptive B cell humoral immunity is absent. They are also over represented in the aged as again this represents a period of waning adaptive immunity (19–22). B regulatory (B_reg_) cells and marginal-zone B (MZ) cells are also classified as ILBs. Importantly, immature and follicular B (FO) cells, while adaptive, also express CD5 and are often present in CD5^+^ B cell preparations, despite being adaptive B cells (17, 18).

Here we report for the first time the finding that the percentage of circulating human B cells that are CD5^+^ is strongly predictive of sensitivity to TCDD-mediated suppression of IgM secretion. Further, isolated CD5^+^ ILB are selectively sensitive to TCDD-mediated AHR activation as evidenced by suppression of IgM secretion, which is not due to IgG class switching, and induction of LCK compared to CD5^-^ B cells. We show that CD5^-^ B cells transiently express low levels of CD5 in response to activation while CD5^+^ ILB remain strongly CD5 positive. We further demonstrate that CD5^+^ and CD5^-^ B cells have similar profiles of activation as evidenced by expression of activation markers CD69, HLA-DR, CD80, and CD86. The differential sensitivity of CD5^+^ ILB to TCDD is due, at least in part, to significantly higher basal expression of AHR and reduced basal expression of AHR repressor, a negative regulator of AHR, in CD5^+^ ILB compared to CD5^-^ B cells. We also show that CD5^+^ ILB basally express higher levels of the immune suppressive receptor, programmed cell death-1 (PD-1) as well as it’s ligands, programmed death ligand 1 (PD-L1) and programmed death ligand 2 (PD-L2). Importantly, we also show that TCDD-mediated AHR activation significantly increases PD-1 protein positivity, suggesting a role for PD-1 in AHR-mediated suppression of IgM secretion. These findings provide critical new insights into AHR-mediated immunotoxicity in human B cells and represent a novel mechanism for xenobiotic-mediated immune suppression.

## Materials and Methods

### Chemicals and Reagents

TCDD (99.1% purity) dissolved in dimethyl sulfoxide (DMSO) was purchased from AccuStandard Inc., (New Haven, CT). Tissue culture grade DMSO was purchased from Sigma Aldrich (St. Louis, MO) and was used to dilute TCDD and also as a vehicle control. AHR antagonist, CH223191 (≥98.0% purity), dissolved in DMSO was purchased from Tocris (Bristol, United Kingdom).

### Human Leukocyte Packs and Purification of Naïve B Cell Subsets

Peripheral blood mononuclear cells (PBMC) collected from anonymous platelet donors were obtained from Gulf Coast Regional Laboratories (Houston, TX). Leukocyte packs that tested negative for HIV, HBV, HCV, and HTLV were shipped at 20–24°C overnight. The next day leukocyte packs were diluted with Hanks Buffered Saline Solution (HBSS) and overlaid on Ficoll-Paque Plus density gradient (GE Healthcare, Piscataway, NJ) and centrifuged for 25 min at 1300xg with low acceleration and low brake. Buffy coats were isolated, washed, counted, and naïve human B cells were enriched using magnetic column-based isolation. B cells were negatively selected for CD19^+^CD27^-^ using MojoSort Human Naïve B cell Isolation kits (Biolegend, San Diego, CA) following the manufacturer’s instructions. Naïve B cell purity was ≥95% as determined by flow cytometric analysis. CD5^+^ B cells were further isolated from CD19^+^ naïve B cells utilizing positive selection. Biotinylated anti-CD5 antibody, clone UCHT2, was incubated with CD19^+^ naïve B cells on ice for 15 mins. Cells were then incubated with Anti-Biotin MicroBeads UltraPure (Miltenyi) for the isolation of minor cellular populations per the manufacturer’s instructions. CD5^+^ B cells were then separated *via* magnetic separation, with CD5^-^ B cells in the decanted cell solution. CD5^+^ B cell purity checks were performed with an altered protocol to visualize CD5 isolated cells *via* flow cytometry. In brief, naïve CD19^+^ B cells were surface stained for 30 mins. with a CD5-PE antibody (clone UCHT2, Biolegend). After the staining period, cells were incubated with a Biotin anti-phycoerythrin (PE) antibody (clone PE001, Biolegend) and then isolated *via* positive selection as described above.

### Cell Culture

Purified B cells were activated with 100 ng/mL soluble CD40 Ligand (Enzo, Farmingdale, NY), 100ng/mL rIL-21 (R&D Systems, Minneapolis, MN) and 1 ng/mL rIL-2 (Roche Applied Science, Indianapolis, IN) and seeded at a density of 1x10^6^ cells/mL in Roswell Park Memorial Institute (RPMI) 1640 medium supplemented with 5% human AB serum (Valley Biomedical, Winchester, VA), 50 μM of 2-mercaptoethanol (ThermoFisher, Lafayette, CO) and 100 U/mL penicillin and 100 μg/mL streptomycin (Life Technologies, Carlsbad, CA). In experiments testing TLR activation of CD5^+^ B cells, TLR agonists tested were TLR2 (Lipotoeic acid), TLR4 (LPS), TLR5 (Flagellin), TLR7 (R837), or TLR9 (CpG). All TLR agonists were purchased from Invivogen (San Diego, CA) and used at a concentration of 10μg/mL. To test PD-1 receptor functionality, human soluble, recombinant PD-1 ligand 1 or PD-1 ligand 2 (Biolegend) was added at the initiation of B cell culture at a concentration of 1 μg/mL (sPD-L1) or 0.1 μg/mL (sPD-L2) per manufacturer’s instructions. Human B cells were treated with 10nM TCDD, 10μM CH223191, or 0.04% DMSO at the time of activation unless otherwise stated. B cells were cultured at 37°C with 5% CO_2_ for various lengths of time depending on the specific measurement. Cells were harvested at indicated times for flow cytometric analysis of protein expression and mRNA analysis. For ELISPOT measurement of IgM secretion, B cells were harvested on day 7 post activation. All data are from TCDD responsive donors as evidenced by >20% TCDD-mediated suppression of IgM compared to vehicle control. Data points were normalized to their own internal vehicle control, as the inter-individual variability in the magnitude of the control IgM response between human donors precludes the ability to determine significant differences between human donors in terms of response to TCDD (23, 24).

### Enzyme-Linked Immunospot Assay (ELISPOT)

In brief, multiscreen 96-well filter plates (Milipore, Burlington, MA) were coated with 5 μg/mL of purified mouse anti-human IgM antibody (Sigma-Aldrich) overnight, washed and blocked with 5% bovine serum albumin (BSA) (Sigma Aldrich) for 2 hours. During this time, B cells were harvested and washed 3x with RPMI 1640, enumerated, and resuspended in supplemented RPMI with 10% bovine calf serum (ThermoFisher) and incubated overnight at 37°C. The following day, filter plates were incubated with biotin-conjugated mouse anti-IgM (Sigma Aldrich) and streptavidin-horseradish peroxidase (Sigma Aldrich) for 1 h at 37°C. All incubations were preceded by 3 washes with phosphate buffered saline plus 0.1% Tween-20 (Sigma Aldrich) and 3 washes with nanopure water. IgM positive spots were developed with aminoethyl carbazole staining kit (Sigma Aldrich). Spots were quantified using Immunospot Software (Cellular Technology, Shaker Heights, OH) and normalized to the number of viable cells plated in each well.

### Enzyme-Linked Immunosorbent Assay (ELISA)

The concentration of IgM and IgG secreted into the culture supernatant was quantified by sandwich ELISA. In brief, Immulon 4 HBX 96-well microtiter plates (VWR International, Radnor, Pennsylvania) were coated with anti-human IgM and IgG antibodies (both 1 μg/ml; Sigma-Aldrich) overnight. Culture media collected from human B cells were incubated over primary antibody-coated plates for 90 min at 37°C with 5% CO_2_ and was followed by overlaying with anti-human IgM-HRP or anti-human IgG-HRP conjugated antibodies (Sigma-Aldrich). Incubations were followed by washes with phosphate-buffered saline (pH 7.4) containing 0.05% Tween-20 (Sigma-Aldrich) and PBS. 2,2’-Azino-bis (3-ethylbenzothiazoline-6-sulphonic acid) (ABTS, Sigma-Aldrich) was then added as a colorimetric substrate for HRP. The rate of colorimetric change was quantified with a Synergy HT microplate reader (BioTek, Winooski, Vermont) at 405 nm for 1 h. Concentrations of IgM and IgG in media were calculated based on a standard curve created in each plate with purified human IgM and IgG standards (Sigma Aldrich).

### Gene Expression Analysis

Human CD19^+^, CD5^+^, or CD5^-^ B cells were taken directly *ex vivo* or activated and treated with 10 nM TCDD. At indicated times, cells were collected and centrifuged at 8500 x g for 5 min. Cell pellets were stored at -80°C. B cell RNA was isolated using RNeasy kits (Qiagen, Hilden, Germany) following manufacturer’s instructions. cDNA libraries were generated by subjecting RNA to reverse transcription using High Capacity cDNA RT-PCR kits (Applied Biosystems, Foster City, CA) with random priming. cDNA was amplified using Taqman Gene Expression assays (Applied Biosystems) with the following primer/probes: *IGHM* (Hs00941538_g1), *AHR* (Hs00169233_m1), *AHRR* (Hs01005075_m1), *CYP1A1* (Hs01054797_g1), *ARNT* (Hs01121918_m1), *PDCD1* (Hs01550088_m1), which encodes PD-1, CD274 (Hs00204257_m1), encoding PD-L1, and PDCD1LG2 (Hs00228839_m1), which encodes PD-L2. All primer/probe sets are commercially available from Applied Biosystems. All quantitative real-time PCR reactions were performed using Applied Biosystems model Quantstudio3 detection system. Human 18s ribosomal RNA (Applied Biosystems) was used to normalize input and monitor nucleic acid quality in response to cell isolation or treatment with TCDD. Fold change in gene expression was calculated using the ΔΔCt method.

### Flow Cytometry

For flow cytometric analysis of target protein expression, B cells were activated and treated as indicated. In brief, ~0.25x10^6^ cells were collected at indicated times and were stained with the following: PE mouse anti-human CD5 clone UCHT2, PerCP-Cy5.5 mouse anti-human CD5 clone UCHT2, PE-Cy7 mouse anti-human CD19 clone HIB-19, PE mouse anti-human PD-1 clone A17188B, PerCP-Cy5.5 mouse anti-human PD-L1 clone 29E.2A3, APC mouse anti-human PD-L2 clone MIH18, and Alexa Fluor 647 anti-human LCK Clone LCK-01 (All from Biolegend) following suggested usage. For evaluation of B cell activation, FITC mouse anti-human CD19 clone HIB-19, PerCP-Cy5.5 mouse anti-human CD80 clone 2D10, APC mouse anti-human CD86 clone BU63, BV421 mouse anti-human HLA-DR clone L243, and BV510 mouse anti-human CD69 clone FN50 (All from Biolegend) were used per manufacturer’s usage. APC anti-human CD40 Antibody clone 5C3 and Brilliant Violet 421 anti-human CD289 (TLR9) Antibody clone S16013D (All from Biolegend) were used to determine CD40 and TLR9 expression. In brief, cells were surface stained on ice for 30 mins and then fixed with CytoFix (BD Bioscience). For intracellular quantification of LCK, cells were surface stained for CD19 for 30 mins on ice, fixed/permeabilized for 20 mins on ice (CytoFix/CytoPerm, BD Biosciences) and then stained intracellularly for LCK for 30 mins on ice per manufacturer’s recommendations. For all experiments, live cells were identified with Fixable Live/Dead Near-IR dye (Life Technologies) and gated on single cell lymphocytes. Positive gates were drawn using unstained and day 0 controls. Cells were processed using a BD FACSCanto II using FACS Diva software (BD Bioscience) and analyzed using FloJo (Version 10, Treestar Software, Ashland OR).

### Institutional Biosecurity

The experiments were conducted in accordance with the Michigan State University the Office of Research Conduct and Biological Safety in an authorized biosafety level 2 laboratory.

### Statistical Analysis

**Figure 1:**
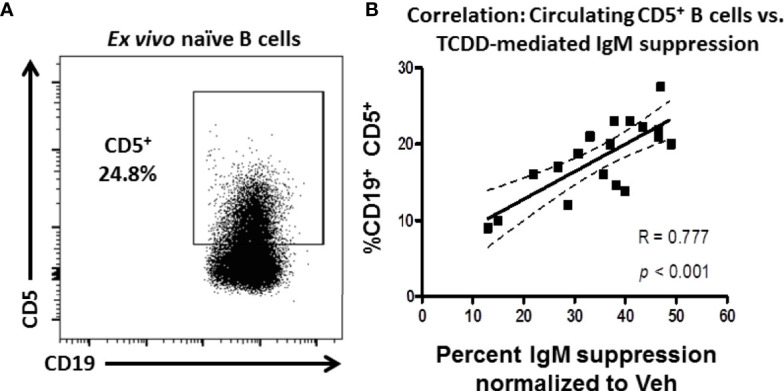
Frequency of CD5 expressing B cells in circulation predicts donor sensitivity to TCDD-mediated IgM suppression. CD19^+^ naïve human B cells isolated from PBMC by magnetic separation were activated with sCD40L, IL-21, and IL-2 and treated with TCDD (10nM) or Veh (0.04% DMSO) as comparator for 7 days. On day 7 cells were collected and either surface stained for CD5 protein expression or assayed for IgM secretion by ELISPOT. The %IgM suppression for a given donor was calculated by normalizing each by the number of IgM^+^ spots in the TCDD-treated samples to the number of spots in each donors Veh control. A representative flow plot of CD19 expressing CD5^+^ B cells is shown in panel **(A)** CD5^+^ cells were identified in the lymphocyte, singlet gate by gating on live CD19^+^ cells. The %IgM suppression was then graphed against the frequency of CD5 expression and is shown in panel **(B)** A linear regression analysis was performed to calculate slope, best fit (R), and significance. Data are from 7 independent experiments assessing a total of 18 human donors.

Linear regression analysis was performed to calculate slope, best fit (R), and significance (**Figure 1B**) using GraphPad Prism software. Unless otherwise stated, significance was calculated using a repeated measures one-way ANOVA with a Tukey’s posttest. A two-way ANOVA was used in **Figures 3**, **4**, and **10**. Significance is indicated by * = p<0.05, ** = p<0.01, and *** = p<0.001. In **Figures 3** and **4**, significance indicated by * represents significant differences to day 0 where * = p<0.05, ** = p<0.01, and *** = p<0.001. “a” indicates significant differences between CD5^+^ and CD5^-^ B cells at same time point where a = p<0.05, aa = p<0.01, and aaa = p<0.001. For **Figure 6**, significance indicated by * represents significant differences to CD40L activation. “a” indicates significant differences to TLR9 activation. *=p<0.05, **=p<0.01, a=p<0.05, and aa=p<0.01. Error bars represent the standard deviation for each group.

## Results

### The Frequency of CD5^+^ B Cells in Peripheral Blood of Human Donors Predicts Their Sensitivity to TCDD-Mediated IgM Suppression

It is well established that TCDD-mediated AHR activation varies from human to human and mouse strain to mouse strain as evidenced by differential suppression of IgM secretion (23, 25). This phenomenon is well defined in mouse strains due to naturally occurring polymorphisms in the AHR receptor (e.g., b and d alleles of the AHR), which affects responsiveness to ligand binding (25). However, other factors could influence the diversity of responses to AHR activation such as differences in the relative frequency of a sensitive cell population. Given that CD5^+^ ILB express LCK and LCK was differentially regulated by TCDD treatment, we hypothesized that the frequency of CD5^+^ B cells in peripheral blood could be predictive of relative sensitivity for any given individual to IgM suppression by TCDD.

To test this possibility, naïve B cells were isolated from the PBMC of 18 human donors. These cells were treated with 10nM TCDD, a high affinity AHR ligand, and activated with CD40L and IL-21 for 7 days for IgM secretion using an IgM ELISPOT or taken directly *ex vivo* and stained for CD19 and CD5 expression. As shown in **Figure 1A**, CD5^+^ B cells were detectable in naïve B cells taken from peripheral blood with the average frequency of CD5 expression was approximately 18%. When the frequency of CD5^+^ CD19^+^ B cells is plotted against the respective suppression of IgM by TCDD in any given donor, we observe a strong, significant, positive correlation between the frequency of CD5 expression and the magnitude of IgM suppression elicited by TCDD treatment compared to Veh control (**Figure 1B**). These results show that TCDD-mediated IgM suppression is due, in part, to the overall levels of CD5 expressing B cells in circulation. Further, our results also suggest that CD5^+^ ILB may have preferential sensitivity to TCDD-mediated AHR activation.

### CD5^+^ ILB Are a Sensitive Human B Cell Population to TCDD-Mediated Suppression of IgM Secretion

**Figure 2:**
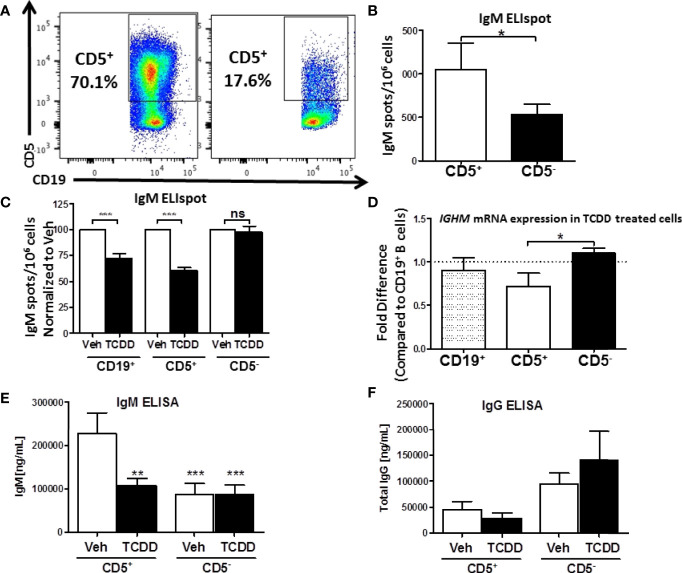
Isolated CD5^+^ B cells secrete more IgM and less IgG compared to CD5^-^ B cells and are preferentially sensitive to TCDD-mediated suppression of IgM secretion. CD19^+^ B cells were isolated from PBMC as previously described and then surface stained for 30 min with anti-CD5-PE antibody. Cells were then incubated with anti-PE biotin beads for 15 min, washed, incubated with strepavidin-ferous beads, and isolated by positive selection based on CD5 expression. Cells were then activated and treated as previously described or surface stained for CD19. Day 0 CD5^+^ and CD5^-^ cell purity is shown in panel **(A)** Cells were gated on live, lymphocyte singlets. Activated CD5^+^ and CD5^-^ cells were cultured for 7 days. After the culture period the relative number of IgM secreting cells was quantified by IgM ELISPOT (panel B). In treated cells, the percent suppression of IgM was calculated by normalizing IgM secretion in TCDD treated cells to each donor’s respective Veh control. For qRT-PCR analysis of *IGHM* mRNA, activated and treated cells were collected on Day 7, lysed, and RNA extracted. cDNA libraries were generated and qRT-PCR performed for *IGHM* and *18s*. The ΔΔCt method was used to calculate relative expression of *IGHM* compared to total CD19^+^ B cells shown in **(D)** Secreted IgM and IgG in culture supernatants were quantified by anti-IgM and anti-IgG ELISAs which are shown in panel **(E, F)**, respectively. A student’s t test was used to determine significance in **(B)** A one-way ANOVA with a Tukey’s posttest was used to determine significance in **(C–F)** While not significant, the p values for comparison of CD19+ B cell *IGHM* mRNA to CD5^+^ and CD5^-^ B cells were p=0.4128 and 0.2075, respectively. Results are from 6 independent experiments assessing a total of 17 human donors **(B–D)** or 4 experiments assessing a total of 8 human donors **(E, F)**. ns = not significant, *p < 0.05, **p < 0.01, and ***p < 0.001.

While the finding that the frequency of circulating CD5^+^ B cells predicts sensitivity to TCDD is impactful, it was not a direct measure of the relative sensitivity of CD5^+^ ILB to TCDD treatment in comparison to non-CD5 expressing B cells. To directly evaluate the sensitivity of CD5^+^ and CD5^-^ B cells to IgM suppression by TCDD, we utilized a double isolation approach, first isolating naïve human B cells from PBMC by negative selection and then enriching for CD5 expression using positive selection for CD5. While the donor to donor frequency of CD5 expressing B cells varied, an average of 18% of naïve B cells from PBMC expressed CD5, demonstrating the relatively small fraction of the overall circulating B cell pool they represent (**Figure 1B**). When we assessed CD5 purity using flow cytometry, we observed that, on average, about 70% of B cells in the CD5^+^ group were CD5 expressing with approximately 30% contamination of non-CD5 expressing cells (**Figure 2A**). The presence of contaminating CD5^-^ cells after CD5^+^ enrichment is not surprising given the difficulty of obtaining a completely pure preparation of a minority cell population. However, in the CD5^-^ group, we achieved a near complete depletion of CD5 expressing cells.

Isolated CD5^+^ and CD5^-^ B cells were treated with 10nM TCDD and activated as previously described for 7 days. After the 7-day culture period we assessed the relative secretion of IgM in Veh treated CD5^+^ and CD5^-^ B cells by ELISPOT. CD5^+^ ILB secreted 2-fold more IgM per million cells when compared to CD5^-^ B cells (**Figure 2B**). Further, when we examined the effect of AHR activation by TCDD on IgM secretion in these cells, we found that TCDD treatment significantly reduced IgM secretion (~30%) in bulk naïve CD19^+^ B cells (**Figure 2C**). Interestingly, TCDD treatment invoked even greater suppression of IgM secretion in CD5^+^ B cells, with an average of a 40% reduction in the overall number of IgM^+^ spots (**Figure 2C**). Surprisingly, CD5^-^ B cells appeared to be refractory to suppression of IgM secretion by TCDD.

We next wanted to verify these findings by quantitative Realtime PCR to assay expression of the IgM heavy chain. Bulk CD19^+^, CD5^+^, and CD5^-^ B cells were isolated, treated, and activated as described above. Following the 7 day culture period, cells were quantified for *IGHM* RNA levels. Similar to previously reported results from our lab, we did not observe a robust decrease in *IGHM* RNA, despite a clear trend towards a decrease (**Figure 2D**). Likewise, while we did not observe any significant differences between *IGHM* mRNA expression in CD19^+^ and CD5^+^ B cells, there was a significant reduction in *IGHM* mRNA in CD5^+^ B cells compared to CD5^-^ (**Figure 2D**). This is likely due to the fact that CD5^+^ B cells make up a relatively small subset of B cells present in the bulk CD19^+^ B cell population. When those cells are depleted as in the CD5^-^ pool, we actually observed a slight increase in *IGHM* mRNA expression in TCDD treated CD5^-^ B cells (**Figure 2D**). Finally, we tested the possibility that AHR-mediated suppression of IgM secretion was due, in part, to IgG class switching. To test this hypothesis, collected culture supernatants from activated CD5^+^ and CD5^-^ B cells stimulated for 7 days in the presence or absence of 10nM TCDD were assessed for IgM and IgG secretion by ELISA. As shown in **Figure 2E**, we observed significantly more IgM secretion in vehicle treated CD5^+^ B cells compared to vehicle treated CD5^-^ B cells, resulting in two-fold more IgM. Further, TCDD treatment of CD5^+^ B cells resulted in decreased IgM secretion, decreasing from ~200μg IgM to ~100μg IgM, an approximately 50% reduction in the concentration of secreted IgM compared to vehicle. However, when we quantified secreted IgG from the same culture supernatants, we observed ~30-fold less secreted IgG from CD5^+^ B cells in comparison to IgM (**Figures 2E, F**). Further, there was no detectable increase in IgG secretion from TCDD-treated CD5^+^ B cells, suggesting that decreased IgM secretion is not due to TCDD-mediated IgG class switching (**Figure 2F**). Curiously, we did observe a trend toward greater IgG secretion in activated, as well as TCDD-treated CD5^-^ B cells compared to CD5^+^, suggesting a greater capacity for IgG secretion from CD5^-^ B cells (**Figure 2F**). Taken together, these results support the finding that CD5^+^ ILB are the primary source of IgM in the absence of infection, and that these cells are directly more susceptible to TCDD-mediated suppression of IgM compared to CD5^-^ B cells, and this suppression is not due to increased IgG class switching.

### CD5^+^ B Cells Have a Similar Profile of Basal and Induced Activation Compared to CD5^-^ B Cells

**Figure 3:**
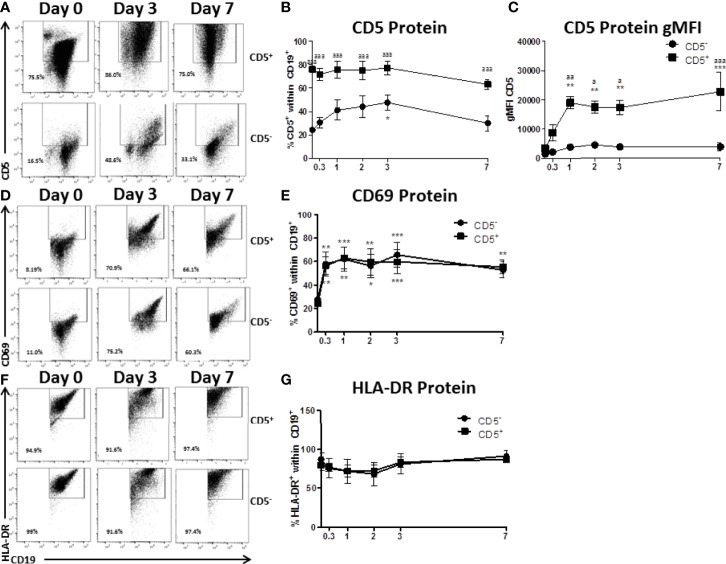
CD5^+^ B cells remain strongly CD5 positive while CD5^-^ B cells transiently acquire CD5 expression despite similar activation-induced profiles of CD69 and HLA-DR. CD19^+^ B cells were isolated from PBMC as previously described and then surface stained for 30 min with anti-CD5-PE antibody. Cells were then incubated with anti-PE biotin beads for 15 min, washed, incubated with strepavidin-ferous beads, and isolated by positive selection based on CD5 expression. CD5 positive and negative cells were activated as previously described or taken at day 0 for purity stain and quantification of CD69 and HLA-DR. At each indicated time point, cells were collected and surface stained for CD19, CD5, CD69, and HLA-DR. For comparison of cell types, all data is on gated CD19^+^ cells within the live lymphocyte gate. Representative flow plots for CD5 expression at select times are shown in **(A)** Cell surface CD5 protein expression over time is shown for all donors in panel **(B)** The geometric mean fluorescence intensities of the per cell basis level of expression for CD5 is shown in panel **(C)** Representative flow plots of CD69 positive cells at select times are shown in **(D)** Cell surface CD69 protein expression over time is shown for all donors in panel **(E)** Representative flow plots of HLA-DR positive cells at select times are shown in **(F)** Cell surface HLA-DR protein expression over time is shown for all donors in panel **(G)** Data shown are from 3 independent experiments assessing a total of 5 human donors. Significant differences compared to day 0 were determined within each cell type by a one-way ANOVA with a Tukey’s posttest where *p < 0.05, **p < 0.01, and ***p < 0.001. Significance between cell types at a given timepoint was determined with a two-way ANOVA with a Tukey’s posttest where ^a^p < 0.05, ^aa^p < 0.01 and ^aaa^p < 0.001.

CD5 is an inhibitory receptor that associates with the antigen receptor of T cells and a subset of B cells and suppresses signaling through the antigen receptor (26). This raised the interesting possibility that CD5^+^ B cells isolated from peripheral blood were merely pre-activated CD5^-^ B cells. To address this possibility, CD5^+^ and CD5^-^ B cells were isolated as described and activated for 7 days with IL-2, IL-21, and CD40 ligand. Over the course of the 7 day activation, cells were collected and monitored for CD5 expression, as well as the activation markers CD69 and HLA-DR, the human MHC class II molecule. As shown in primary flow plots (**Figure 3A**), magnetic cell enrichment resulted in highly enriched populations of CD5^+^ and CD5^-^ B cells where the average purity in both populations was between ~75 – 80%. Following B cell activation, CD5 protein positivity on the surface of CD5^+^ B cells was relatively stable across the 7-day activation as no significant changes compared to day 0 were observed. However, CD5^-^ B cells did modestly increase CD5 protein positivity in response to B cell activation (**Figure 3A**), which was significantly elevated by day 3, increasing from ~20% on day 0 to 40% by day 3 (**Figure 3B**). However, the frequency of CD5^+^ B cells decreased to levels similar to those seen on day 0, suggesting a transient increase of CD5 protein by CD5^-^ B cells in response to activation while CD5 positivity was maintained by CD5^+^ B cells (**Figure 3B**). In fact, CD5 expression was significantly different between the two cell populations at every time point assessed, including day 7 (**Figure 3B**). Next, when we examined the expression of CD5 protein on a per cell basis using the geometric mean fluorescence intensity (gMFI), we observed stark differences in the levels of expression of CD5. For example, the gMFI for CD5 on CD5^+^ cells in both populations was similar (~2000). However, following activation, there was a ten-fold increase in CD5 gMFI on CD5^+^ cells, with an average gMFI of ~20000 within 24 hours and continued to increase until day 7 (**Figure 3C**). Conversely, the gMFI of CD5 within the CD5^-^ population increased from 2000 to 5000 by day 2; however, the increases in CD5 gMFI in CD5^-^ cells were not significant compared to day 0 at any of the time points tested (**Figure 3C**). Of note, CD5 expression continued to increase on CD5^+^ cells through day 7 (**Figure 3C**). When we assessed CD69 positivity by CD5^+^ and CD5^-^ B cells we found minimal CD69^+^ cells expressed by either cell type basally, with between 10 – 20% CD69^+^ cells directly *ex vivo* from peripheral blood (**Figures 3D, E**). After stimulation, both cell types rapidly increased CD69 protein positivity, with a significant 3-fold increase within the first 8 hours of activation (**Figure 3E**). Both CD5^+^ and CD5^-^ B cells maintained CD69 positivity throughout the time course. Conversely, CD5^-^ B cells began modestly decreasing CD69 by day 7 as indicated by a lack of significance compared to day 0 CD69^+^ cells (**Figure 3E**). A similar profile of activation was obtained by assessing HLA-DR expression on CD5^+^ and CD5^-^ B cells, where both populations were mostly positive and maintained positivity with a similar kinetic profile through day 7 (**Figures 3F, G**).

**Figure 4:**
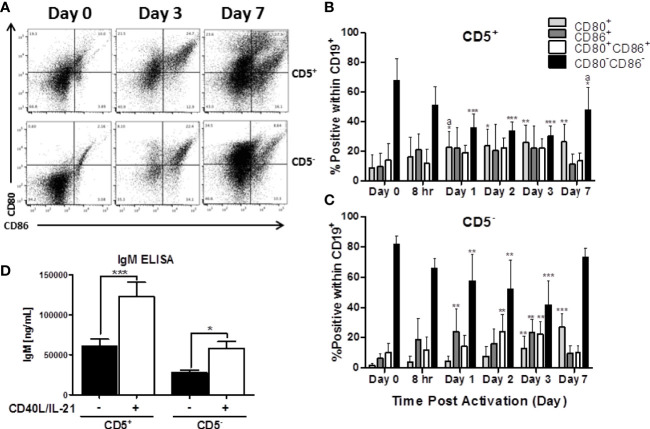
Both CD5^+^ and CD5^-^ are negative for markers of activation, CD80 and CD86, directly *ex vivo*, which is increased following activation with CD40L and IL-21. CD5^+^ and CD5^-^ B cells were isolated and activated as previously described, taken at day 0 for purity stain and quantification of CD80 and CD86, or cultured in complete RPMI supplemented with IL-2 alone. At each indicated time point, cells were collected and surface stained for CD19, CD5, CD80, and CD86. For comparison of cell types, data is on gated CD19^+^ cells within the live lymphocyte gate. Representative flow plots for CD80 and CD86 expression at select times is shown in **(A)**. Cell surface CD80^+^, CD86^+^, CD80^+^CD86^+^ or CD80^-^CD86^-^ protein expression over time is shown for CD5^+^ B cells in panel **(B)** and CD5^-^ B cells in panel **(C)**. Secreted IgM from CD5^+/-^ B cells +/- IL-21 and CD40L are shown in panel **(D)**. Data shown are from 3 independent experiments assessing a total of 5 human donors. Significant differences compared to day 0 were determined within each cell type by a one-way ANOVA with a Tukey’s posttest where *p < 0.05, **p < 0.01, and ***p < 0.001. Significance between cell types at a given timepoint was determined with a two-way ANOVA with a Tukey’s posttest where ^a^p < 0.05. Significance for IgM accumulation in culture supernatants between activated and non-activated cells was determined with a one-way ANOVA with a Tukey’s posttest where *p < 0.05 and ***p < 0.001.

Another critical set of activation markers expressed by B cells is CD80 and CD86. The functionally similar proteins are expressed by activated B cells, macrophage, and antigen-presenting cells and facilitate their interactions with T cells *via* binding to CD28 and CTLA-4 (27). As such, we quantified the expression of CD80 and CD86 by CD5^+^ and CD5^-^ B cells both *ex vivo* and after activation. As shown in representative flow plots in **Figure 4A**, the majority of both CD5^+^ and CD5^-^ lack expression of CD80, CD86, or both directly *ex vivo* with ~70 and 80% of CD5^+^ and CD5^-^ B cells being CD80^-^CD86^-^ respectively (**Figures 4B, C**). After activation, both B cell populations increase expression of CD80 and CD86 with both CD5^+^ and CD5^-^ B cells having significant reductions in the CD80^-^CD86^-^ double negative population within the first 24 hours and was maintained through the first 3 days of B cell activation (**Figures 4B, C**). By day 7 post-activation, expression of CD80 and CD86 began to decrease on CD5^-^ B cells as indicated by an increase in the CD80^-^CD86^-^ double negative population (**Figure 4C**). Interestingly, while the trend was similar in CD5^+^ B cells, there were two key differences: a) CD5^+^ B cells had significantly more CD80 positive cells within the first 24 hours of activation compared to CD5^-^ B cells; and b) CD5^+^ B cells had a slower decrease in the percent positive cells for CD80 and CD86 by day 7 when compared to CD5^-^ B cells (**Figure 4B**). As both B cell populations did have some level of CD80 and CD86 expression *ex vivo*, we next determined whether the isolation procedure appeared to be stimulatory. As shown in **Figure 4D**, both CD5^+^ and CD5^-^ B cells secreted detectable levels of IgM when cultured for 7 days in the absence of IL-21 and CD40 ligand, with CD5^+^ B cells secreting ~2 fold more than CD5^-^ B cells. However, both B cell populations increased IgM secretion markedly following activation, with both increasing IgM secretion ~2.5 fold (**Figure 4D**). Taken together, these data suggest that CD5^+^ and CD5^-^ B cells have similar activation profiles both *ex vivo* and following stimulation *in vitro*. Both cell populations have equivalent levels of basal expression and kinetic profiles for CD69, HLA-DR, and CD80 and CD86, while only CD5^+^ B cells maintain high levels of CD5 expression after activation when compared to CD5^-^ B cells. While the possibility remains that some fraction of the CD5^+^ population is accounted for by activated, CD5^-^ B cells, these data suggest that that explanation is insufficient to fully explain the functional and phenotypic differences observed between these two B cell populations.

### TCDD-Mediated AHR Activation Induces LCK Expression in CD5^+^ Human B Cells

Previous work from Zhou and colleagues showed that TCDD-mediated increases in LCK accompanied IgM suppression (8); however, those studies were performed in bulk CD19^+^ naïve human B cells. We next wanted to determine if the TCDD effect on LCK expression was confined to the CD5 expressing B cells, or if it occurred in CD5^-^ B cells as well. To this end, we isolated CD5^+^ and CD5^-^ human B cells from PBMC as previously described. These cells were treated with TCDD, activated with CD40L and IL-21 and cultured for 7 days. On day 3, an aliquot of cells was collected for assessment of LCK expression by flow cytometry, a time point previously identified as showing increased LCK expression by TCDD treatment. Sensitivity to TCDD-mediated suppression of IgM secretion was confirmed on the remaining CD5^+^ cells using an IgM ELISPOT on day 7 of culture (data not shown).

**Figure 5:**
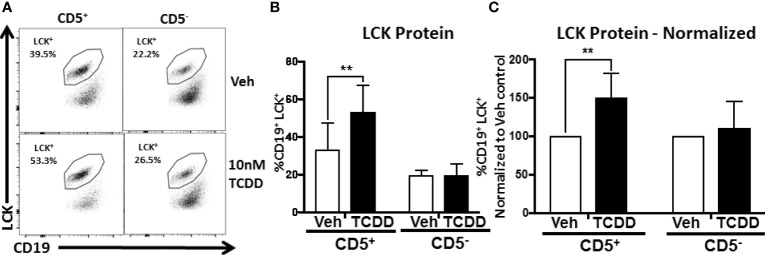
TCDD-mediated AHR activation increases LCK expression in CD5^+^ but not CD5^-^ human B cells. CD19^+^ naïve B cells were isolated from PBMC and further enriched into CD5^+^ and CD5^-^ B cell populations. Cells were then activated and treated with 10nM TCDD as described previously. Cells were collected on day 3 post activation and surface stained for CD19, permeabilized and incubated with an anti-LCK antibody for quantification of intracellular LCK protein expression by flow cytometry. Representative flow plots are shown in panel **(A)**. LCK^+^ cells were identified in the lymphocyte singlet gate by gating on live CD19^+^ cells. Averaged results from 3 independent experiments assessing a total of 9 human donors are shown in **(B)**. Expression of LCK protein normalized to Veh control is shown in **(C)**. A repeated measure one-way ANOVA with a Tukey’s posttest was used to determine significance. **p < 0.01.

We detected modest LCK expression in Veh treated CD5^+^ B cells as well as CD5^-^ B cells to a lesser extent (**Figure 5A**). However, when CD5^+^ B cells were treated with TCDD, we detected an increase in LCK expression similar to previously reported studies (**Figure 5A**) (8). As shown in **Figure 5B**, the average frequency of LCK^+^ CD5^+^ ILB was between 30 and 35% on average in the absence of TCDD treatment. Conversely, CD5^-^ B cells expressed between 15 and 20% LCK (**Figure 5B**). In CD5^+^ B cells, TCDD treatment resulted in significant upregulation of LCK protein expression, increasing it by approximately 1.5-fold to 50-55% (**Figure 5B**). In contrast, TCDD treated CD5^-^ B cells expressed nearly identical LCK protein levels compared to Veh treated controls (**Figure 5B**). When we normalized LCK protein expression in TCDD treatment groups to their respective Veh controls, we observed similar results. TCDD treatment increased LCK protein expression in CD5^+^ B cells by 150% but CD5^-^ B cells were refractory to this effect as indicated by no change in LCK protein expression (**Figure 5C**). These results support the hypothesis that CD5^+^ ILB are preferentially sensitive to TCDD treatment as the previously reported effect of TCDD on LCK expression was confined exclusively to the CD5^+^ B cell pool.

### Human CD5^+^ ILB Respond Equally to Adaptive and Innate Stimuli

**Figure 6:**
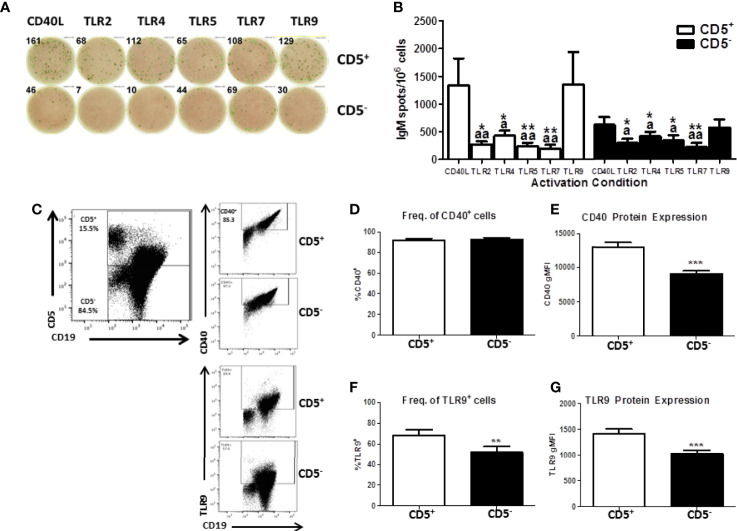
Human CD5^+^ B cells respond to T-dependent and T-independent activators. Human CD19^+^ naïve B cells were isolated from PBMC and separated into CD5^+^ and CD5^-^ populations as previously described. Cells were then activated with IL-21, IL-2, and either CD40L or indicated TLR agonist. After the 7-day culture period, cells were collected and IgM secretion assessed *via* IgM ELISPOT. For quantification of CD40 and TLR9, freshly isolated CD19^+^ B cells were stained for CD19, CD5, and CD40 surface expression. Cells were then fixed and permeabilized and stained intracellularly for TLR9. Representative ELISPOT wells from a given donor are shown in panel **(A)** Averaged results from 3 independent experiments assessing a total of 8 human donors are shown in panel **(B)** Representative flow plots showing CD5, CD40, and TLR9 are shown in panel **(C)** Averaged results from 2 independent experiments assessing a total of 9 human donors are shown in panels **(D–G)**. A repeated measures ANOVA with a Tukey’s posttest was used to determine significance in panel B where * indicates significant differences compared to CD40L activation within each cell type. “a” indicates significant differences compared to TLR9 activation within each cell type. *p < 0.05, **p < 0.01, ^a^p < 0.05, and ^aa^p < 0.01. A paired t-test was used to determine significance in panels **(D–G)** where **p < 0.01 and ***p < 0.001.

While we have demonstrated a selective effect of TCDD-mediated AHR activation in CD5^+^ B cells, we wanted to verify that activation with CD40L was optimal given the innate-like nature of these cells. To test this possibility, CD5^+^ and CD5^-^ B cells were cultured with IL-21 and CD40L or toll-like receptor (TLR) stimulation. TLRs tested were TLR 2 (lipotoeic acid), TLR4 (LPS), TLR5 (flagellin), TLR7 (R837), or TLR9 (CpG). As shown in **Figure 6A**, CD5^+^ B cells responded well to CD40L and CpG activation. On average, CD40L activated CD5^+^ B cells produced 1000 IgM^+^ spots per 1 million cells as quantified by ELISPOT (**Figure 6B**). TLR2, 5, and 7 stimulation of CD5^+^ B cells resulted in minimal IgM secretion (**Figure 6B**). Stimulation of CD5^+^ B cells with CpG resulted in a robust IgM response nearly identical to CD40L (**Figure 6B**). TLR4 stimulation did induce IgM secretion in CD5^+^ ILB, but it was much more modest compared to CD40L and CpG with only about 500 IgM^+^ spots per million cells (**Figure 6B**). Interestingly we observed an identical response profile in CD5^-^ B cells, with CD40L and CpG inducing the greatest IgM response (**Figure 6B**). However, we again noted that CD5^-^ B cells produced about half of the IgM that CD5^+^ ILB do, when stimulated with either CD40L or CpG (**Figure 6**). We next determined if the differences in responsiveness to CD40L or CpG by CD5^+^ B cells was due to differences in expression of CD40 or TLR9. Freshly isolated CD19^+^ B cells were stained for CD5, CD40, and intracellularly TLR9. As shown in **Figure 6C**, CD40 and TLR9 were readily detectable on both CD5^+^ and CD5^-^ B cells. However, despite a similar frequency of CD40^+^ cells between CD5^+^ and CD5^-^ B cells, CD5^-^ B cells expressed significantly less CD40 on their cell surface as evidenced by a ~30% reduction in CD40 gMFI (**Figures 6D, E**). Unlike CD40, there was significantly less TLR9^+^ CD5^-^ B cells when compared to CD5^+^ B cells (**Figure 6F**). Moreover, when TLR9 expression was quantified, we found that CD5^-^ B cells expressed significantly less TLR9 on a per cell basis compared to CD5^+^ B cells (**Figure 6G**). These data show that CD5^+^ B cells express more CD40 and TLR9 compared to CD5^-^ B cells, suggesting a putative explanation for the differences observed in IgM secretion in response to CD40L and CpG. Consequently, the frequency of TLR9^+^ cells was also greater in the CD5^+^ B cell population, further suggesting critical phenotypic differences between CD5^+^ and CD5^-^ B cells. Together these findings would argue against the possibility that CD40L activation is fundamentally inappropriate in CD5^+^ ILB. Indeed, it has been reported in the literature that despite their innate-like profile, CD5^+^ ILB can and do participate in adaptive B cell germinal center reactions (11).

### Differential Sensitivity of Human CD5^+^ B Cells to TCDD Is Due to Increased Expression and Activity of AHR

Next, experiments were undertaken to gain an understanding why human CD5^+^ B cells were selectively sensitive compared to CD5^-^ B cells. As discussed previously, TCDD is a prototypical ligand for AHR, and studies have correlated the relative expression levels of AHR to the responsiveness of cells to the toxic effects of TCDD (28). To test this possibility, human B cells were isolated from PBMC as previously described and further isolated into CD19^+^ bulk B cells, CD5^+^ B cells, or CD5^-^ B cells. Cells were then lysed directly and RNA extracted and qRT-PCR performed for *AHR*, *ARNT* (Aryl Hydrocarbon Receptor Nuclear Translocator encoding gene), *AHRR* (Aryl Hydrocarbon Receptor Repressor), and *CYP1A1*, the gene which encodes the cytochrome p450 1A1 xenobiotic metabolizing enzyme, a surrogate of AHR activity (29) or activated with CD40L and IL-21 to determine the effects of B cell activation on gene expression at indicated time points.

**Figure 7:**
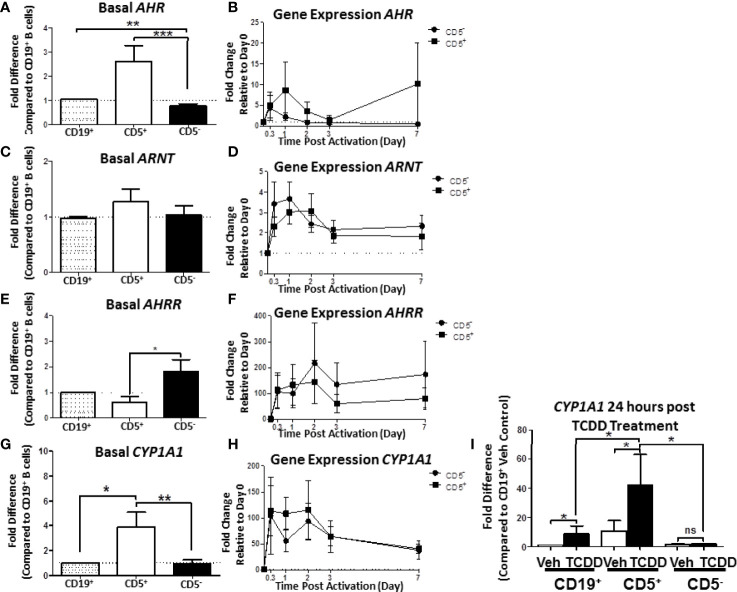
Human CD5^+^ B cells express higher basal *AHR* and *CYP1A1* and reduced basal *AHRR* compared to CD5^-^ B cells. CD19^+^, CD5^+^, and CD5^-^ B cells were isolated as previously described and either lysed for RNA extraction, activated, or treated with 10nM TCDD for 24 hours. Activated cells were collected at the indicated times, and cell pellets were stored at -80°C until RNA extraction. Cells treated with TCDD were lysed and RNA extracted after the 24-hour treatment. Extracted RNA was reverse transcribed into cDNA libraries and the relative gene expression for *AHR*, *ARNT*, *AHRR*, and *CYP1A1* was determined by qRT-PCR. For panels **(A, C, E, G)**, relative gene expression was compared to CD19^+^ bulk B cells. In panels **(B, D, F, H)**, relative gene expression was determined compared to Day 0. For panel **(I)**, *CYP1A1* gene expression was compared to CD19^+^ B cells treated with Veh. Results are from 3 independent experiments assessing a total of 8 human donors. Significance was calculated using a repeated measures ANOVA with a Tukey’s posttest. ns = not significant, *p < 0.05, **p < 0.01, and ***p < 0.001.

When compared, the relative levels of AHR mRNA expression in human CD5^+^ B cells were approximately 2.5- to 3-fold greater than in CD5^-^ B cells (**Figure 7A**). Moreover, CD5^-^ B cells expressed significantly less AHR mRNA even in comparison to the CD19^+^ bulk B cell population, suggesting that the CD5^+^ population was responsible for the majority of basal AHR expression indicated in the bulk B cell population (**Figure 7A**). When activated, both CD5^+^ and CD5^-^ B cells increased their relative *AHR* mRNA levels compared to day 0 (**Figure 7B**); however, there was significant donor-to-donor variability in the fold induction. The trend in the level of increase was comparable between the two cell populations within the first 8 hours of activation with CD5^-^ B cells rapidly decreasing expression levels while CD5^+^ B cells continued to increase expression of *AHR* mRNA in the first 24 hours (**Figure 7B**). When we compared the relative basal expression of *ARNT*, which codes for an accessory protein that facilitates AHR transcriptional activity *via* heterodimerization in the nucleus, we did not observe any significant differences in relative expression in any of the B cell populations (**Figure 7C**). Following activation, *ARNT* mRNA levels increased in both cell populations. Unlike the trend in AHR mRNA expression, CD5^-^ B cells increased *ARNT* expression more rapidly than CD5^+^ B cells within the first 24 hours of activation, with both populations having equivalent *ARNT* mRNA levels by 48 hours post-activation (**Figure 7D**). Further, when we quantified basal expression of a negative regulator of AHR, AHRR, we found that CD5^+^ B cells expressed significantly reduced basal levels of *AHRR* mRNA when CD5^+^ and CD5^-^ B cells were compared to CD19^+^ B cells (**Figure 7E**). Similarly, following activation, CD5^-^ B cells trended toward higher induction of *AHRR* mRNA within the first 48 hours of activation compared to CD5^+^ B cells, with this trend maintained over the course of the 7 day activation (**Figure 7F**). Given that the CD5^+^ B cells expressed decreased *AHRR* mRNA and increased *AHR* mRNA, the activation status of AHR was assessed by the relative expression of the AHR-regulated gene, *CYP1A1*. As shown in **Figure 7G**, CD5^+^ B cells expressed significantly more *CYP1A1* mRNA compared to both CD19^+^ bulk B cells and CD5^-^ B cells, with an average of a 4-fold increase in relative expression. As the levels of AHR increased post B cell activation, we also determined whether *CYP1A1* gene expression also increased after B cell activation. As shown in **Figure 7H**, we observed comparable levels of *CYP1A1* gene expression within the first 8 hours of B cell activation. However, similar to the trend in *AHR* gene expression, CD5^-^ B cells quickly decreased *CYP1A1* gene expression while remaining stable in CD5^+^ B cells 48 hours post-activation (**Figure 7H**). While the variability in the kinetics of gene induction precluded statistical significance, these data show that not only do CD5^+^ B cells constitutively express higher levels of AHR basally but it is also functionally active as evidenced by AHR-dependent gene transcription.

While human B cells are a sensitive immunological target of TCDD-mediated AHR activation, they express much less *CYP1A1* in response to TCDD as compared to hepatocytes (30). The degree of basal CYP1A1 expression observed in CD5^+^ B cells was much higher than what has been reported in CD19^+^ bulk B cells (31), so we next sought to test if their AHR could be further activated by TCDD treatment alone. To test this possibility, B cells were isolated into CD19^+^, CD5^+^, and CD5^-^ populations as previously described. Cells were treated with TCDD for 24 hours. After the treatment period, cells were assessed for *CYP1A1* mRNA expression. As shown in **Figure 7I**, TCDD treatment significantly increased *CYP1A1* expression in CD19^+^ B cells showing a ~9-fold increase compared to its Veh control. However, as observed previously, CD5^+^ B cells also expressed ~9-fold more *CYP1A1* basaly compared to Veh treated CD19^+^ B cells (**Figure 7I**). Consequently, after 24 hours of TCDD treatment, *CYP1A1* mRNA expression increased precipitously, culminating in an approximately 40-fold increase compared to CD19^+^ B cells (**Figure 7I**). Surprisingly, CD5^-^ B cells did not increase *CYP1A1* mRNA expression in response to TCDD treatment, further suggesting that these cells are refractive to TCDD-mediated AHR activation. Indeed these data suggest that the preferential sensitivity of CD5^+^ B cells to TCDD-mediated AHR activation is due, in part, to higher expression of AHR, and decreased expression of AHRR. Further, AHR appears to be active within these cells, even in the absence of an activating ligand such as TCDD, yet they retain the capacity to further respond to AHR activation as evidenced by the increase in *CYP1A1* mRNA expression after TCDD treatment.

### Human CD5^+^ B Cells Have a Regulatory Profile as Evidenced by Increased Expression of PD-1, PD-L1, and PD-L2

While we found that human CD5^+^ ILB expressed significantly more AHR compared to CD5^-^ B cells as an explanation for their selective sensitivity to an AHR ligand, it does not explain how AHR activation suppresses their ability to secrete IgM. While less work has been conducted on human CD5^+^ ILB, murine B1 B cells have been extensively studied. It has been reported that CD5^+^ B1 B cells from extralymphatic sites (i.e., tonsil) express more PD-1 and its ligands compared to adaptive B cells from spleen or lymph nodes (32, 33). PD-1 is the prototypical immune inhibitory receptor that functions by dampening antigen receptor signaling on target cells (Reviewed in (34)). It is well established in T cells that PD-1 functions as part of the normal regulation of ongoing adaptive T cell responses (35) but the role of PD-1 expression by B cells is less characterized.

**Figure 8:**
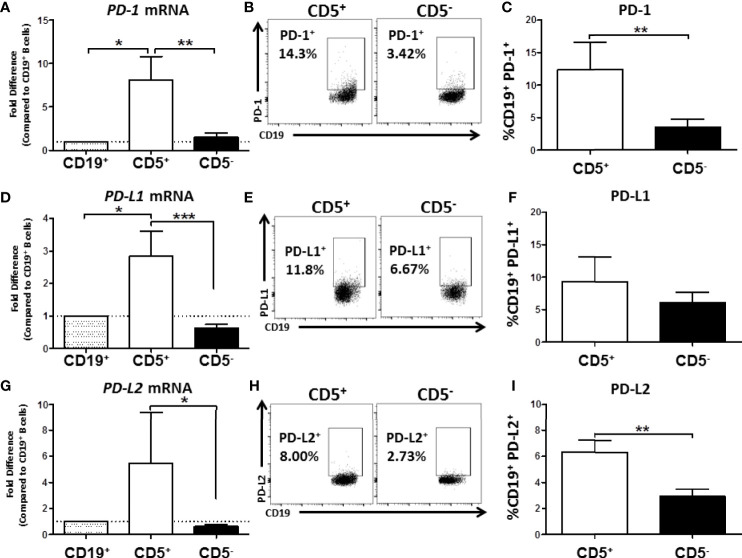
Human CD5^+^ B cells express higher basal PD-1, PD-L1, and PD-L2 compared to CD5^-^ B cells. CD19^+^, CD5^+^, and CD5^-^ B cells were isolated as previously described and either lysed for RNA extraction or surface stained for PD-1, PD-L1, and PD-L2 protein expression directly *ex vivo*. Extracted RNA was reverse transcribed into cDNA libraries and the relative gene expression for *PDCD1*, *CD274* and *PDCD1LG2* was determined by qRT-PCR. For panels **(A, D, G)** relative gene expression was compared to CD19^+^ bulk B cells. Representative flow plots for PD-1, PD-L1, and PD-L2 protein expression is shown in panels **(B, E, H)**. PD-1^+^, PD-L1^+^, and PD-L2^+^ cells were identified in the lymphocyte, singlet gate by gating on live CD19^+^ cells. Averaged results from 3 independent experiments assessing a total of 8 human donors for PD-1, PD-L1, and PD-L2 is shown in panels **(C, F, I)**. Significance was calculated using a repeated measures ANOVA with a Tukey’s posttest. *p < 0.05, **p < 0.01, and ***p < 0.001.

As part of our initial characterization of human CD5^+^ ILB, we wanted to assess expression of PD-1 mRNA and protein levels. Naïve B cells from PBMC were separated into CD19^+^ bulk B cells, CD5^+^, or CD5^-^ B cells as previously described. Cells were then either lysed for RNA extraction and qRT-PCR or incubated with anti-CD19 and PD-1 antibodies for quantification of surface protein expression. As shown in **Figure 8A**, we found that CD5^+^ B cells expressed approximately 8-fold more *PDCD1* (the gene that encodes PD-1) mRNA compared to both CD19^+^ bulk B cells and CD5^-^ B cells. This finding was confirmed when we examined PD-1 cell surface protein expression. As shown in **Figure 8B**, PD-1 was detectable on both CD5^+^ and CD5^-^ B cells; however, we found a 4-fold increase in the percentage of CD5^+^ B cells with PD-1 protein expressed on the cell surface compared to CD5^-^ B cells (**Figure 8C**).

Next we assessed the expression of *CD274* and *PDCD1LG2*, the genes for PD-L1 and PD-L2, respectively. As with PD-1 mRNA expression, we found that CD5^+^ B cells expressed significantly more PD-L1 and PD-L2 mRNA compared to CD5^-^ B cells resulting in 3- and 5-fold higher levels, respectively (**Figures 8D, G**). When we examined protein levels of both ligands on the cell surface, a similar trend was observed wherein a greater percentage of CD5^+^ B cells expressed PD-1 ligand protein compared to CD5^-^ B cells (**Figures 8E, H**). However, PD-L2 cell surface protein expression was observed on a significantly greater percentage of CD5^+^ B cells compared to CD5^-^ B cells across experiments, with CD5^+^ B cells expressing 2x more on average (**Figure 8I**), whereas there were no statistically significant differences in the percentage of PD-L1^+^ cells between CD5^+^ and CD5^-^ B cells (**Figure 8F**). This could be explained, in part, by the ubiquitous expression of PD-L1, with many immune cells expressing this protein (36). PD-L2 is hypothesized to be more restricted in its pattern of expression, primarily relegated to antigen presenting cells and regulatory cell subsets (36). Together, these findings suggest that as with murine B1 B cells, human CD5^+^ B cells have a regulatory immune profile as evidenced by their expression of PD-1 and its ligands.

### TCDD-Mediated AHR Activation Induces Increased Percentage of Human CD5^+^ ILB With PD-1 Protein but Not CD5- B Cells

**Figure 9:**
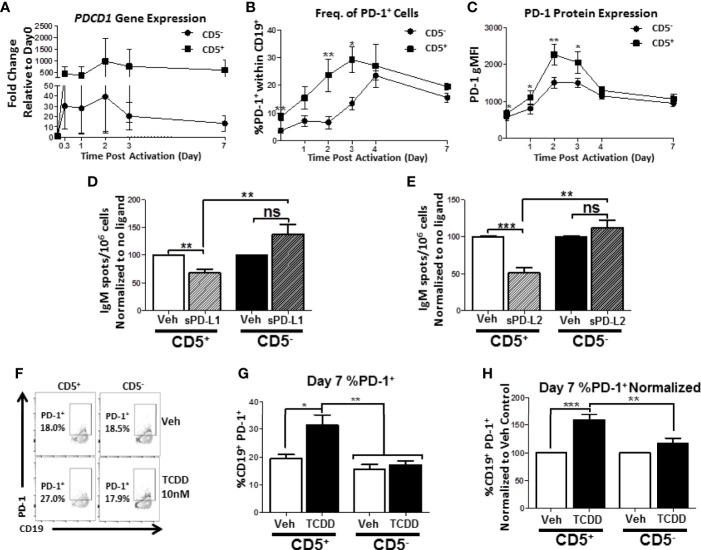
Human CD5^+^ B cells display enhanced PD-1 expression kinetics compared to CD5^-^ B cells and TCDD treatment results in a significant increase in day 7 CD5^+^ PD-1^+^ B cells. Human CD5^+^ and CD5^-^ B cells were isolated, activated, and, where indicated, treated with sPD-L1 (1μg/mL), sPD-L2 (0.1μg/mL) or TCDD (10nM) for 7 days as previously described. At the indicated times, cells were collected for either RNA analysis for *PDCD1* mRNA expression or surface stained with anti-PD-1 antibody and the frequency of cell surface PD-1 protein expression quantified by flow cytometry. In panel **(A)**, the fold change in *PDCD1* mRNA at indicated times compared to day 0 is shown, corresponding to 4 independent experiments assessing a total of 7 human donors. PD-1^+^ cells were identified in the lymphocyte singlet gate by gating on live CD19^+^ cells with the kinetics of PD-1 protein expression following B cell activation is shown in panels **(B, C)** corresponding to 2-4 independent experiments analyzing 4-8 human donors. Averaged, normalized IgM ELISPOT results from sPD-L1 and sPD-L2-treated CD5^+^ and CD5^-^ B cells assessing 11 human donors corresponding to 4 independent experiments is shown in panels **(D, E)**, respectively. Representative flow plots for Veh and TCDD treated CD5^+/-^ B cells are shown in panel **(F)**. Averaged frequency of PD-1^+^ cells, both raw and normalized to Veh control, are shown in panels **(G, H)** from 2 independent experiments assessing a total of 6 human donors. Significance in panels **(D, E, G, H)** was determined by a repeated measures two-way ANOVA with a Tukey’s posttest. ns=not significant, *p < 0.05, **p < 0.01, and ***p < 0.001. A paired, repeated measures t-test was used to determine significance at each indicated time post-activation **(B, C)** where *p < 0.05 and **p < 0.01.

We have shown that human CD5^+^ ILB are preferentially sensitive to TCDD-mediated suppression of IgM and that CD5^+^ ILB express significantly more PD-1 and its ligands. Given these findings, the potential for PD-1 involvement in facilitating TCDD-mediated immune suppression is strong. Therefore we hypothesized that TCDD-mediated AHR activation regulates PD-1 protein expression on CD5^+^ ILB. First, we defined the kinetics of *PDCD1* mRNA and PD-1 protein expression in response to B cell activation in the absence of AHR activation. CD5^+^ and CD5^-^ B cells were activated as previously described and during the 7 day culture period, cells were collected at the indicated times and quantified for both *PDCD1* mRNA and surface PD-1^+^ cells by flow cytometry. As shown in **Figure 9A**, both CD5^+^ and CD5^-^ B cells increased *PDCD1* mRNA expression within 8 hours of B cell activation with CD5^+^ B cells increasing *PDCD1* mRNA by 300 – 500 fold while CD5^-^ B cells increased by 30 – 50 fold (**Figure 9A**). Although the levels of *PDCD1* gene expression were stable within the first 48 hours of B cell activation in both populations, CD5^-^ B cell *PDCD1* mRNA began declining while it remained stable in CD5^+^ B cells (**Figure 9A**). Studies in T lymphocytes have suggested that PD-1 protein expression is significantly induced by days 2 and 3 post-activation and begins to wane by day 4/5 post-activation (37, 38). Likewise, studies in murine B1 cells have also suggested that both B1-a and B1-b B cells from peritoneum, blood, and spleen also show detectable induction of PD-1 protein expression beginning on day 2 after activation and then start to decline by day 5 (39). As shown in **Figure 9B**, we detected minimal PD-1^+^ cells in resting CD5^-^ B cells, with less than 5% of cells expressing PD-1. Conversely, we detected two-fold more PD-1^+^ cells in resting CD5^+^ B cells (**Figure 9B**). Upon B cell activation, we observed a robust and steady increase in the frequency of PD-1 protein expression on CD5^+^ B cells, which peaked on day 3 (**Figure 9B**). Interestingly, CD5^-^ B cells displayed a delayed pattern of PD-1 upregulation in response to B cell activation, where the frequency of PD-1^+^ cells did not begin to increase until about day 3 post activation and continued to increase (**Figure 9B**). Further, when we assessed the levels of PD-1 protein expressed, we observed similar kinetics, where CD5^+^ B cells expressed significantly more PD-1 protein on their cell surface in comparison to CD5^-^ B cells in the first 3 days after B cell activation (**Figure 9C**). However, by day 4, both cell populations expressed similar levels of PD-1 protein (**Figure 9C**). We then assessed PD-1 receptor function to determine if PD-1 ligation suppressed IgM responses on either CD5^+^ or CD5^-^ B cells. To test this possibility, freshly isolated CD5^+^ and CD5^-^ B cells were activated and treated with recombinant human PD-L1 or PD-L2 for 7 days. Following the culture period, cells were collected and IgM secretion was measured by IgM ELISPOT. As shown in **Figure 9D**, when CD5^+^ B cells were treated with sPD-L1, we observed a significant 30% decrease in the number of IgM^+^ spots. Interestingly, we did not observe significant modulation of IgM secretion by CD5^-^ B cells treated with sPD-L1 (**Figure 9D**). Similarly, sPD-L2 treatment also significantly reduced IgM secretion in CD5^+^ but not CD5^-^ B cells, resulting in a ~50% decrease in the number of IgM^+^ spots compared to CD5^+^ B cells receiving no ligand (**Figure 9E**).

We next quantified the effect of TCDD-mediated AHR activation on PD-1 protein expression by activated CD5^+^ and CD5^-^ B cells. As shown in **Figures 9F, G**, the percentage of cells expressing surface PD-1 by day 7 was equivalent between CD5^+^ and CD5^-^ B cells. However, when CD5^+^ ILB were treated with TCDD, there was a significant ~10% increase in the percentage of CD5^+^ B cells with detectable PD-1 protein on the cell surface by day 7 post-activation, which represented a 60% increase in the frequency of PD-1 receptor protein expression (**Figures 9F, G, H**). Strikingly, we observed no effect on day 7 in PD-1 receptor expression by CD5^-^ cells after TCDD-treatment (**Figures 9F, G, H**). Collectively, these results suggest that PD-1 is differentially regulated in CD5^+^ compared to CD5^-^ B cells as evidenced by their kinetics of expression in the absence of AHR activation. Further, we demonstrate that PD-1 receptor ligation by either PD-1 ligand suppresses IgM secretion by CD5^+^ but not CD5^-^ B cells. Finally, the finding that TCDD-treatment increased the frequency of PD-1^+^ CD5^+^ B cells strongly implicate the regulation of PD-1 by AHR activation as a potential mechanism for TCDD-mediated suppression of human CD5^+^ B cells.

### TCDD-Mediated AHR Activation Enhances Cell Surface PD-1 Protein Expression on CD5^+^ B Cells, but Is Not Required for PD-1 Expression

While the results obtained in **Figure 9** indicated that TCDD-mediated AHR activation could regulate PD-1 expression on CD5^+^ B cells, we wanted to directly quantify the contribution of AHR activation to PD-1 expression as we observed differences with activation alone. As such, we employed a well characterized AHR antagonist, CH223191 (40), added concurrently with TCDD to block AHR activation in CD5^+^ and CD5^-^ B cells. As we are working with human B cells, the levels of AHR vary from donor to donor. Likewise, CD5^+^ B cells express elevated levels of AHR compared to CD5^-^ B cells (**Figure 7A**) and as such, we used a thousand-fold more antagonist (10 μM) compared to TCDD (10 nM) to ensure AHR activity is blocked. To this end, CD5^+^ and CD5^-^ B cells were isolated, treated with either TCDD or AHR antagonist, and activated as previously described. PD-1 cell surface protein expression was quantified by flow cytometry on days 2 and 3, time points which corresponded to the upregulation of PD-1 on CD5^+^ B cells (**Figure 9B**).

**Figure 10:**
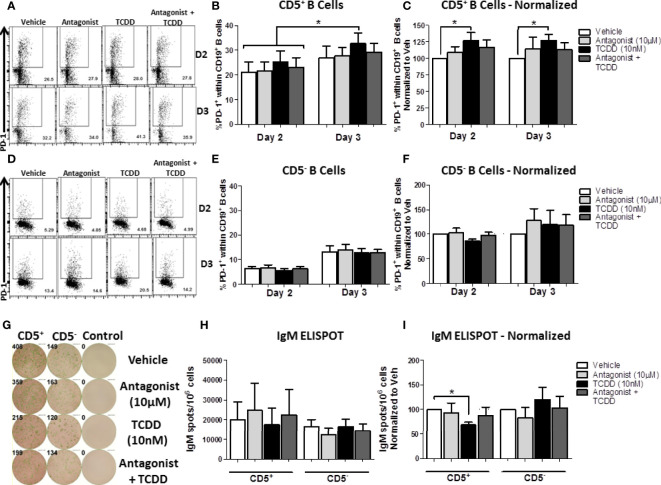
TCDD-mediated AHR activation significantly induced PD-1 protein expression in CD5^+^ ILB by days 2- and 3-post activation and is reduced by treatment with AHR antagonist, CH223191. Human CD5^+/-^ B cells were isolated from human PBMC as previously described. Cells were then treated with either DMSO, 10 μM CH223191, 10 nM TCDD, or TCDD plus antagonist, and activated as described for 7 days. Cells were collected on days 2 and 3 post-activation for quantification of cell surface PD-1 protein levels. Representative flow plots from each time point/treatment/cell type are shown in panels **(A, D)**. Averaged raw percentage expression of PD-1 within gated CD19^+^ B cells are shown in panels **(B, E)** with data normalized to the respective vehicle controls shown in panels **(C, F)**. Cells were also collected at day 7 post-activation for functional analysis by IgM ELISPOT. Representative select ELISPOT wells are shown in panel **(G)**. Averaged raw IgM spots per million cells are shown for each cell type and treatment group in panel **(H)** and spots normalized to each respective vehicle control are shown in panel **(I)**. Data are from 4 independent experiments assessing a total of 8 human donors. Significance was determined using a two-way, repeated measures, ANOVA with a Tukey’s posttest. *p < 0.05.

As shown in **Figure 10A**, PD-1 expression was readily detectable on CD5^+^ B cells by day 2 post-activation, and continued to increase by day 3. While there was donor to donor variation in the induction of PD-1 protein expression, 20% of CD5^+^ B cells expressed PD-1 by day 2 on average, which increased to ~35% by day 3 (**Figure 10B**). When we compared the effect of TCDD treatment on PD-1 expression, we found significant increases in PD-1 positivity by TCDD treated CD5^+^ B cells at both days 2 and 3-post activation, corresponding to a 25% and 30% increase, respectively, when compared to vehicle treated controls (**Figure 10C**). While blocking TCDD-mediated AHR activation with antagonist did reduce PD-1 frequencies, they were not reduced to vehicle levels (**Figure 10C**). Interestingly, antagonist-treated CD5^+^ B cells did not express reduced PD-1 frequencies below those seen on vehicle treated controls, with some demonstrating enhanced PD-1 positivity (**Figure 10C**), suggesting that AHR is not the primary regulator of PD-1 expression.

Similarly to CD5^+^ B cells, CD5^-^ B cell PD-1 expression was detectable by day 2 post-activation and increased robustly by day 3 post-activation (**Figure 10D**). Unlike CD5^+^ B cells, the percentage of CD5^-^ B cells expressing PD-1 at either time point was remarkably consistent (**Figure 10E**). TCDD-mediated AHR activation in CD5^-^ B cells did not appear to influence PD-1 expression at either day 2 or 3-post activation (**Figure 10F**). Likewise, AHR antagonist alone or in combination with TCDD treatment did not significantly alter the expression of PD-1 protein (**Figure 10F**) suggesting that AHR does not regulate PD-1 in CD5^-^ B cells. To confirm that the AHR antagonist blocked AHR activation, we quantified the IgM responses from CD5^+^ and CD5^-^ B cells on day 7 post-activation. As shown in **Figure 10G**, we observed robust secretion of IgM after activation with CD5^+^ B cells secreting more IgM on average (**Figure 10H**). When the effect of TCDD treatment on IgM secretion was quantified, we observed significant suppression of IgM secretion in CD5^+^ but not CD5^-^ B cells by TCDD (**Figure 10I**) and treatment with AHR antagonist restored the IgM response (**Figure 10I**), which demonstrated that AHR antagonist treatment blocked TCDD-mediated AHR activation. Together, these data suggest that TCDD-mediated AHR activation in CD5^+^ B cells can regulate PD-1 protein expression; however, AHR alone is not responsible for regulating PD-1 expression by CD5^+^ ILB.

## Discussion

In the current study we report for the first time the finding of a differentially sensitive human B cell subpopulation to AHR-mediated immune suppression, CD5^+^ ILB. We determined that CD5^+^ B cells were likely not a preactivated B cell population in the peripheral blood by showing a similar profile of immune activation between CD5^+^ and CD5^-^ B cells as well as induction of effector function, i.e., IgM secretion in response to CD40L and IL-21 stimulation. We confirmed the previous finding of AHR-mediated regulation of LCK expression in CD5^+^ but not CD5^-^ B cells (8). The differential sensitivity of CD5^+^ B cells is due, in part, to reduced expression of AHRR and greater levels of AHR expression, which appears active based on *CYP1A1* expression even in the absence of TCDD-mediated activation. Finally, CD5^+^ ILB are marked by increased mRNA expression of the immune inhibitory receptor, PD-1, as well as by both of its ligands. Further, there were significantly more PD-1^+^ and PD-L2^+^ CD5^+^ B cells, and CD5^+^ B cells were preferentially sensitive to PD-1 ligation *in vitro*, suggesting a role for PD-1 in AHR-mediated suppression of IgM. Consequently, we also found that AHR activation by TCDD in CD5^+^ ILB, but not CD5^-^ B cells, significantly upregulated the PD-1 receptor 2, 3, and 7 days post TCDD treatment. However, blocking AHR with an AHR antagonist did not fully reduce the frequency PD-1^+^ cells to or below those found in vehicle treated controls, suggesting AHR can regulate PD-1 expression, but is not required.

Cellular immunology is a rapidly evolving field with new immune cell subsets being identified continuously. While the paradigm of subsetting adaptive immune cells into naïve, effector, and memory subsets has existed for decades, the identification of less frequent, exotic, subsets of immune cells with highly specialized immune functions dominates the field of contemporary immunology. Indeed, our finding of high basal AHR expression as a marker of human CD5^+^ ILB is novel; however, it is not the first reported instance of AHR being described as a critical transcription factor for a novel immune cell population. The endogenous activity of AHR was first described as being important for the generation and effector functions of T_helper_ 17 (Th17) cells *via* the regulation of retinoic acid receptor-related orphan receptor-γt (ROR-γt) (41, 42). Described in 2008 by Kimura and colleagues, AHR was proposed as being necessary for the differentiation of Th17 cells and its activation promoted their ability to secrete IL-17 (43). That same year Funatake and colleagues proposed a similar role for AHR in the generation and maintenance of regulatory T cells and their secretion of IL-10 (44). By 2011, AHR had been described as being functionally critical for the generation of intestinal innate lymphoid cells by several groups (45–47). Since these findings, much focus has been given to how AHR shapes cell fate decisions, and licenses immune cell subsets to exert specific effector profiles, such as the secretion of IL-17/22 (3, 48, 49).

While most of this effort has focused on T lymphocytes, new subsets of human B cells have also been identified, with CD5^+^ ILB being one such example (16). It has been well understood in murine immunology that innate-like B cells are primarily found in the fetal liver, the peritoneal cavity, and the adenoids and these cells are termed B1 B cells. These cells can be further subdivided into B1a, B1b, Breg, B10 (IL-10 expressing B1b cells), and MZ B cells (11, 12, 14, 15, 17, 18). B1 B cells are primarily responsible for secreting polyvalent nIgM to non-T-dependent antigens as well as regulating inflammation through the expression of IL-10, PD-1 ligands, and the removal of pro-inflammatory molecules such as cellular debris (17, 18). Interestingly, high levels of AHR expression have been described in these cells by reports from various groups (50–52). For example, Villa and coworkers described AHR expression levels in several subsets of murine B cells, reporting the highest levels of AHR expression in MZ and CD5^+^ B1a B cells from the peritoneum (51). Indeed, the first reported role of AHR in the regulation of B1 B cells came in 1995 from Fernandez-Salguero and colleagues in which they described a significant loss of CD5^+^ peritoneal B cells in mice that were genetically deficient in AHR (53). These reports directly showed the requirement for functional AHR in maintaining optimal B1 B cell numbers in the mouse. Despite clear evidence within the mouse that CD5^+^ ILB are a distinct, heterogeneous B cell lineage, the exact surrogate in human has not been identified, and currently, the use of CD5 in human peripheral B cells to identify ILB is debated within the field of B cell immunology. While we do not claim to have accurately identified these cells in human, our results do demonstrate the remarkable similarity between murine CD5^+^ B1 B cells and human CD5^+^ B cells from peripheral blood. Further, we report that based on quantified activation markers, CD5^+^ B cells from peripheral human blood do not appear to be just an activated subset of adaptive B cell, but rather a distinct, heterogeneous B cell population. Indeed our findings reported here are one of the first to describe a similar role for AHR in a human CD5^+^ B cell population, and reinforces the notion that AHR has critical roles in shaping immune responses in the absence of exposure to xenobiotics.

What has yet to be elucidated is the role of xenobiotics in regulating these cells following an exposure event. Our findings strongly suggest that further AHR activation in CD5^+^ B cells suppresses their ability to secrete IgM. In the context of ILB effector function, this would have far reaching consequences for human health, such as increased risk of infection by bacterial pathogens, particularly in young children and the elderly where a larger proportion of the B cell repertoire is dominated by ILB (19–22). While most human data regarding exposures to AHR activating xenobiotics are in healthy adults, there are data sets which have focused specifically on children following an exposure event, with the Dutch polychlorinated biphenyl (PCB)/Dioxin study being one. Two of the pertinent findings from the Dutch PCB/Dioxin study were that: (a) Children had increased incidence of otitis media; and (b) Children responded more poorly to vaccination as indicated by antigen specific antibody titers (54). Otitis media is inflammation of the middle ear and is primarily due to bacterial infection, with *Streptococcus pneumoniae* accounting for the majority of cases (55). Critically, nIgM opsonization of pneumococcus is considered an indispensable mechanism of early control of the bacteria, and decreased circulating levels of nIgM have been implicated in allowing for ascension of the bacteria up the eustachian tubes into the middle ear (55–58). Given that children exhibited increased incidences of otitis media following exposure to PCBs and dioxins, it is tempting to speculate that this increase was due, in part, to AHR activation and consequently decreased nIgM producing ILB.

In the same cohort of children, investigators also noted decreased antibody titers following vaccination. While adaptive B cells are primarily responsible for generating humoral immunity following vaccination, it is noteworthy that CD5^+^ ILB are also capable of partaking in germinal center reactions (11). For example, Seifert and coworkers identified two distinct populations of CD5^+^ ILB in human blood; CD27 bearing and CD27 negative (11). CD27 is a surface marker on B cells that denotes memory and germinal center reacted B cells (59). Indeed, when CD5^+^ CD27^-^ ILB gene expression was compared to CD5^+^ CD27^+^ ILB, there was a clear gene signature associated with germinal center reacted cells in the CD27^+^ population (11). Given this possibility, one explanation for the decreased vaccine titers in children exposed to dioxin-like compounds could be due to diminished participation of CD5^+^ ILB in germinal centers, which resulted in poor vaccine outcome.

Alternatively, decreased vaccine titers in children exposed to dioxin-like compounds could be an indirect effect of AHR activation in CD5^+^ ILB *via* the regulation of PD-1 ligands. Our finding that CD5^+^ B cells basally expressed significantly more PD-ligand could have significant immunological consequences for PD-1 bearing cells if AHR could also enhance PD-ligand expression. For example, PD-L2 has been shown to be the more critical than PD-1 ligand in the regulation of antibody responses to non-T-dependent antigens such as pneumococcal polysaccharide (36, 60). McKay and coworkers found that co-administration of a PD-L2, but not PD-L1 blocking antibody increased anti-pneumococcal IgM titers in mice vaccinate with pneumovax, the 13-valent pneumococcal vaccine (60). If TCDD-mediated AHR activation also significantly affected the levels PD-ligand protein expression, TCDD-exposed CD5^+^ ILB would theoretically be able to suppress any PD-1 expressing immune cells they contact, including T cells, which are a critical contributor to germinal center reactions and optimal antibody titers following vaccination (61, 62) putatively explaining decreased vaccine efficacy observed in children exposed to dioxin-like compounds.

While much focus has been placed on the role of AHR in immune cell differentiation and their effector functions, less has been placed on the role of AHR in the regulation of the PD-1 signaling axis. The PD-1 signaling axis has historically been studied in the context of cancer and T cell biology, with less emphasis placed on B cells (34, 63). Indeed, there is a dearth of studies in T lymphocytes, which have examined the question of AHR regulation in the expression of PD-1. For example, Liu and coworkers described the regulation of PD-1 receptor expression on CD8^+^ T cells by kynurenine, an endogenous AHR ligand, secreted by solid tumor cells (64). Indeed, subsequent studies have suggested that AHR cooperation with the RelA subunit of nuclear factor kappa-light-chain-enhancer of activated B cells (NF-κB) regulates PD-1 expression through an excess of NF-κB p50 subunit homodimer formation; a transcription factor which can bind the promoter of the PD-1 gene *PDCD1* (65, 66). A similar mechanism has been proposed for AHR regulation of PD-1 expression in myeloid cells as well (67). While this report is the first to describe a role for AHR in the regulation of PD-1 in B cells, studies from our group and others have demonstrated that TCDD-mediated AHR activation negatively regulates NF-κB activation in B cells. This could be explained, in part by AHR binding with the RelA subunit, which would have negative overall effects on NF-κB activation while facilitating increased expression of PD-1 (65–67). Alternatively, a recent report by Bally and colleagues suggested that the lysine-specific histone demethylase 1 (LSD1) protein was recruited by B lymphocyte-induced maturation protein-1 (BLIMP-1) to repress transcription of PD-1 by binding to the PD-1 promoter region (68). This was confirmed by the finding that in LSD1 deficient mice, CD8^+^ T cells expressed elevated levels of PD-1 (68). It is well known that TCDD-mediated AHR activation significantly suppresses BLIMP-1 expression in human B cells (1). This could suggest that AHR regulates PD-1 expression in CD5^+^ ILB *via* a similar mechanism but this possibility will need to be tested directly. However, studies utilizing an AHR antagonist suggested that the AHR effect on PD-1 receptor expression is insufficient to fully explain our findings. To this end, it is noteworthy that AHR-activation strongly drives expression of LCK in CD5^+^ B cells. A handful of studies going back to 1991 have suggested LCK could potentially regulate PD-1 signaling, primarily *via* IL-2 receptor signaling (69, 70). A recent report by Arulraj and coworkers leveraged machine learning and mathematical modeling to further suggest a role for LCK in PD-1 signaling (71). These findings are consequential as this study as well as the study by Zhou et al. both demonstrated significant upregulation of LCK in B cells; CD5^+^ B cells specifically (8). Given that we did not find that AHR was sufficient on its own to regulate PD-1 protein expression, these findings could suggest that the AHR-mediated effects on LCK expression are potentially more consequential in facilitating IgM suppression.

Lastly, AHR activation has also been shown to regulate the expression of PD-L1 (72). In a recent report by Wang and colleagues, it was shown that *in vitro* and *in vivo* treatment with benzo(α)pyrene (BαP), a carcinogen from tobacco smoke and AHR ligand, resulted in significant expression of PD-L1 (72). Further, when AHR binding elements in the PD-L1 promoter were deleted or cells were treated with an AHR antagonist, BαP failed to induce PD-L1 expression, demonstrating a critical role for AHR. As mentioned prior, CD5^+^ ILB can participate in germinal center reactions with antigen specific T cells as well as adaptive B cells. This leads us to speculate on a scenario in which following an exposure to an AHR ligand, CD5^+^ ILB could be transformed into ‘suppressor’ cells, where they not only suppress their own immune function, but suppress PD-1 bearing cells around them. Wang and colleagues reported a similar finding involving the suppression of CD8^+^ T cell activation by PD-1 bearing B cells (73). When the effect of AHR-activation on PD-1 receptor expression is taken in context with AHR-mediated increases in LCK and the potential to also induce PD-1 ligand expression, one can quickly begin to appreciate how these 3 effects by AHR could converge, resulting in IgM suppression even in the absence of PD-1 downregulation following treatment with an AHR antagonist. Focus on the effects of AHR activation in CD5^+^ ILB in regard to the PD-1/LCK signaling axis will be needed to further elucidate this possibility.

In summary, we have shown that the frequency of circulating CD5^+^ B cells in the total B cell pool is strongly predictive of a human donor’s response to TCDD-mediated immune suppression as evidenced by impairment of IgM secretion. Further, we demonstrate that CD5^+^ B cells are preferentially sensitive to AHR-mediated suppression of IgM secretion compared to CD5^-^ B cells, and this preferential sensitivity is due, in part, to increased basal expression of AHR as well as decreased basal AHRR expression in CD5^+^ B cells. Human CD5^+^ B cells are marked by increased expression of the immune suppressive receptor PD-1 and its ligands, and TCDD-mediated AHR activation increased the frequency of PD-1^+^ CD5^+^ B cells, suggesting a role for PD-1 signaling in AHR-mediated suppression of IgM secretion.

## Data Availability Statement

The original contributions presented in the study are included in the article/supplementary material. Further inquiries can be directed to the corresponding author.

## Author Contributions

LB, JZ, RC, and NK contributed to the development of the experimental design and interpretation of results. LB and JZ conducted the experiments and LB wrote the paper with editing by JZ, RC, and NK. All authors contributed to the article and approved the submitted version.

## Funding

This work was supported by NIH grant P42 ES004911.

## Conflict of Interest

The authors declare that the research was conducted in the absence of any commercial or financial relationships that could be construed as a potential conflict of interest.
